# Ruxolitinib for Emergency Treatment of COVID‐19–Associated Cytokine Storm: Findings From an Expanded Access Study

**DOI:** 10.1111/crj.70050

**Published:** 2025-04-08

**Authors:** Jeffrey Weinstein, Nikhil Jagan, Shawnta Lorthridge‐Jackson, J. E. Hamer‐Maansson, Peg Squier

**Affiliations:** ^1^ Department of Clinical Quality and Infection Prevention and Control Kettering Health Network Dayton Ohio USA; ^2^ Department of Medicine Creighton University School of Medicine Omaha Nebraska USA; ^3^ Medical Affairs Incyte Corporation Wilmington Delaware USA

**Keywords:** acute respiratory distress syndrome, COVID‐19, cytokine release syndrome, expanded access, hospitalization, Janus kinase inhibitors

## Abstract

**Introduction:**

This expanded access program (EAP) provided ruxolitinib (oral, selective Janus kinase [JAK]1/JAK2 inhibitor) for emergency treatment of COVID‐19–associated cytokine storm in patients eligible for hospitalization (NCT04355793).

**Methods:**

Patients received ruxolitinib 5 mg twice daily (preferred regimen when tolerated) or once daily for ≤ 14 days, or until determination of no clinical benefit was made. Outcomes were clinical status, physician‐assessed clinical benefit, and serious adverse event (SAE) incidence.

**Results:**

Of 312 patients, 45.5% achieved ≥ 1‐point clinical status improvement. Physician‐assessed clinical benefit was reported in 42.6% of evaluable patients. SAEs occurred in 42.9%, with 2.6% experiencing an SAE suspected to be ruxolitinib related.

**Conclusions:**

Overall, some hospitalized patients with COVID‐19–associated cytokine storm who received ruxolitinib experienced clinical status improvement; ruxolitinib was well tolerated.

**Trial Registration:**

ClinicalTrials.gov identifier: NCT04355793

## Graphical abstract

In this expanded access program providing ruxolitinib for the emergency treatment of COVID‐19–associated cytokine storm in patients eligible for hospitalization, ruxolitinib 5 mg twice daily (preferred regimen when tolerated) improved clinical status by ≥ 1 point in nearly half of the patients. Ruxolitinib was generally well tolerated, with no thrombotic serious adverse events reported. Randomized controlled trials are warranted to further assess clinical benefit and identify patients with severe COVID‐19 infection who respond to Janus kinase inhibition.

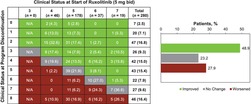

## 1 Introduction

Severe COVID‐19 complications include respiratory failure, acute respiratory distress syndrome (ARDS), sepsis, and multiorgan failure [1]. Cytokine storm results from overproduction of interleukin (IL)‐6 and other cytokines following rapid and excessive immune activation [2], contributing substantially to mortality [3, 4]. IL‐6 and other cytokines elevated in severe COVID‐19 are regulated by the Janus kinase (JAK)/signal transducer and activator of transcription pathway [3].

Ruxolitinib is an oral, selective JAK1/JAK2 inhibitor with clinical activity in hemophagocytic lymphohistiocytosis, a hyperinflammatory syndrome with a macrophage‐derived cytokine profile similar to that observed in COVID‐19 [5, 6, 7]. An expanded access program (EAP) of ruxolitinib for emergency treatment of COVID‐19–associated cytokine storm was initiated (NCT04355793); efficacy and safety findings are presented here.

## 2 Methods

This open‐label, multicenter EAP of ruxolitinib for the treatment of SARS‐CoV‐2–infected US patients with severe cytokine storm enrolled patients ≥ 12 years old with hospitalization‐eligible COVID‐19–associated cytokine storm (see Methods S1 for additional details). Key exclusion criteria were pregnancy or breastfeeding, inadequate liver function, platelet count < 50 × 10^9^/L, concomitant use of another JAK inhibitor, eligibility for another therapeutic clinical study for cytokine storm, or any underlying condition posing unacceptable risk to the patient. Institutional review boards of participating centers approved the protocol.

Patients received ruxolitinib 5 mg twice daily (bid) via oral or nasogastric administration for 7–14 days. Dose reduction to 5 mg once daily (qd) was indicated for patients with platelet counts 50–100 × 10^9^/L and either moderate renal or any level of hepatic impairment. If renal or hepatic impairment improved and platelet count improved to > 100 × 10^9^/L, ruxolitinib could be increased to 5 mg bid. Ruxolitinib 5 mg qd was recommended for patients on dialysis. Patients could receive any nonprohibited medication needed to manage COVID‐19 or complications, including anti‐infectives.

The primary objective was to provide ruxolitinib to hospitalization‐eligible patients with COVID‐19–associated cytokine storm. The secondary objective was to monitor serious adverse events (SAEs) from the time of consent until 30 days after end of treatment; toxicities resolved, returned to baseline, or were deemed irreversible (whichever was longest); or the patient transitioned to a commercial product. SAEs requiring medical or surgical intervention were considered medically significant. The treating physician assessed SAE relatedness to ruxolitinib.

Clinical status was assessed using a 9‐point scale (Table S1). Physicians indicated the clinical status at screening, treatment initiation, during treatment, and at end of treatment. Physicians also reported whether the patient derived clinical benefit from treatment (yes/no).

### 2.1 Statistical Analyses

The number of patients was not predetermined. Agreement between physician‐assessed clinical benefit and clinical status improvement was assessed using McNemar's test for 2 × 2 tables. Other data were summarized descriptively.

## 3 Results

### 3.1 Patients

As of May 24, 2021, 312 patients received ruxolitinib (5 mg bid, *n* = 280; 5 mg qd, *n* = 31; other, *n* = 1). Median (range) age was 67.0 (21–97) years; 179 patients (57.4%) were men; most had a baseline clinical status of 5 (*n* = 190 [60.9%]); and median (range) time to treatment after diagnosis was 6.0 (1–44) days (Table S2). Most patients received starting (*n* = 280 [89.7%]) and final (*n* = 220 [70.5%]) ruxolitinib doses of 5 mg bid; 27 (8.7%) received a final dose of 5 mg qd; the final dose of the remaining 65 (20.8%) could not be confirmed. Median (range) duration of therapy was 7 (0–14) days.

### 3.2 Efficacy

**FIGURE 1:**
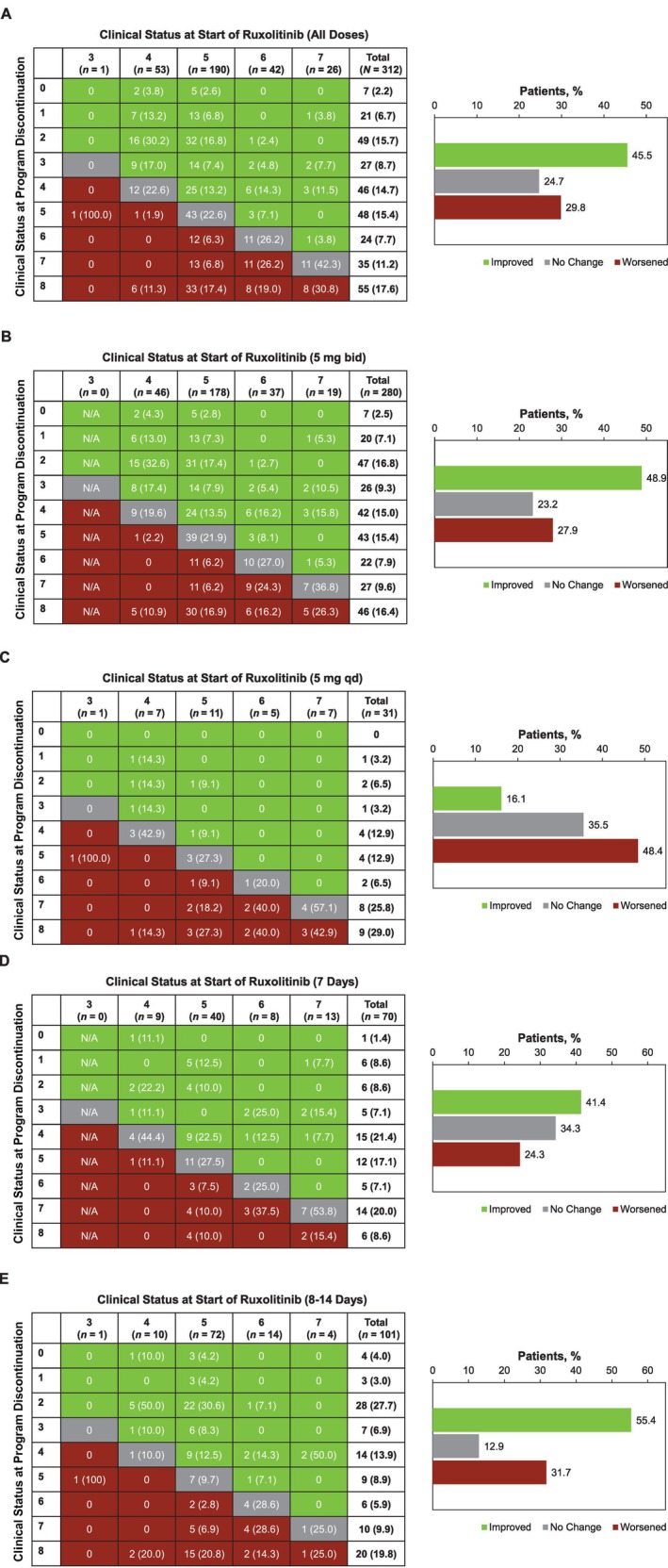
Change in clinical status from start of ruxolitinib treatment to program discontinuation. bid, twice daily; N/A, not applicable; qd, once daily. Data are provided for the overall population (A), by starting ruxolitinib dose of 5 mg bid (B) or 5 mg qd (C), and by duration of treatment of 7 days (D) or 8–14 days (E). Clinical status was based on a 9‐point ordinal scale and defined as follows: (0) uninfected—no clinical or virologic evidence of infection; (1) ambulatory, no limitation of activities; (2) ambulatory—limitation of activities; (3) hospitalized, mild disease, no oxygen therapy; (4) hospitalized, mild disease, oxygen by mask or nasal prongs; (5) hospitalized, severe disease, noninvasive ventilation or high‐flow oxygen; (6) hospitalized, severe disease, intubation and mechanical ventilation; (7) hospitalized, severe disease, ventilation + additional organ support—vasopressors, renal replacement therapy, extracorporeal membrane oxygenation; (8) death.

Among all 312 patients, 142 (45.5%) achieved ≥ 1‐point improvement in clinical status from treatment start to program discontinuation; 77 (24.7%) and 93 (29.8%) experienced no change or a worsening in clinical status, respectively (Figure 1A). Of 280 patients with a starting dose of 5 mg bid, 137 (48.9%) had ≥ 1‐point clinical improvement (Figure 1B); 5/31 (16.1%) receiving a starting dose of 5 mg qd improved (Figure 1C). Of 70 patients who received treatment for 7 days, 29 (41.4%) had ≥ 1‐point clinical improvement (Figure 1D), whereas 56/101 (55.4%) of those who received treatment for 8–14 days had ≥ 1‐point clinical improvement (Figure 1E).

Physicians reported that 42.6% (103/242) of ruxolitinib‐treated evaluable patients experienced clinical benefit (Figure S1). Among evaluable patients receiving starting doses of 5 mg bid and qd, 97/216 (44.9%) and 6/25 (24.0%), respectively, had physician‐assessed clinical benefit. The agreement between physician‐assessed clinical benefit and clinical status improvement among evaluable patients was not statistically significant.

### 3.3 Safety

Overall, 134 patients (42.9%) experienced ≥ 1 SAE (Table 1); the most common was acute respiratory failure (*n* = 40 [12.8%]). ARDS SAEs were only observed in the 5 mg bid group (*n* = 18 [6.4%]). Eight patients (2.6%) had SAEs suspected to be treatment‐related (Table S3). The only treatment‐related SAEs occurring in > 1 patient were septic shock (*n* = 5 [1.6%]) and anemia (*n* = 2 [0.6%]). In total, 28 patients (9.0%) experienced ≥ 1 medically significant SAE (Table S4); the most common was acute kidney injury (*n* = 5 [1.6%]).

**TABLE 1:** SAEs reported in > 1 patient. ARDS, acute respiratory distress syndrome; bid, twice daily; qd, once daily; SAE, serious adverse event.

Characteristic, *n* (%)	Ruxolitinib 5 mg bid (*n* = 280)	Ruxolitinib 5 mg qd (*n* = 31)	All (*N* = 312)^a^
Any SAE	116 (41.4)	18 (58.1)	134 (42.9)
Acute respiratory failure	36 (12.9)	4 (12.9)	40 (12.8)
Respiratory failure	27 (9.6)	3 (9.7)	30 (9.6)
COVID‐19 pneumonia	17 (6.1)	2 (6.5)	19 (6.1)
ARDS	18 (6.4)	0	18 (5.8)
Acute kidney injury	10 (3.6)	2 (6.5)	12 (3.8)
Septic shock	5 (1.8)	5 (16.1)	10 (3.2)
Cardiac arrest	6 (2.1)	1 (3.2)	7 (2.2)
Sepsis	5 (1.8)	1 (3.2)	6 (1.9)
Multiple organ dysfunction syndrome	4 (1.4)	1 (3.2)	5 (1.6)
COVID‐19	4 (1.4)	0	4 (1.3)
Pneumonia	4 (1.4)	0	4 (1.3)
Pneumothorax	4 (1.4)	0	4 (1.3)
Anemia	2 (0.7)	1 (3.2)	3 (1.0)
Arrhythmia	1 (0.4)	2 (6.5)	3 (1.0)
Hypoxia	3 (1.1)	0	3 (1.0)
Cardiogenic shock	1 (0.4)	1 (3.2)	2 (0.6)
Disease progression	2 (0.7)	0	2 (0.6)
Hypotension	1 (0.4)	1 (3.2)	2 (0.6)
Pneumonia aspiration	2 (0.7)	0	2 (0.6)
Pneumonia bacterial	2 (0.7)	0	2 (0.6)
Pneumonia staphylococcal	2 (0.7)	0	2 (0.6)

^a^ Total includes one patient (79‐year‐old woman with baseline clinical status of 5 and treatment initiation 2 days following diagnosis) who did not receive ruxolitinib 5 mg qd or bid; the patient did not experience any SAEs.

## 4 Discussion

This EAP suggests that JAK inhibition with ruxolitinib improves clinical status for some patients with COVID‐19–associated cytokine storm and was generally well tolerated. Nearly half achieved ≥ 1‐point improvement in clinical status, and a similar percentage experienced physician‐assessed clinical benefit. Generally, 5 mg bid was more effective than 5 mg qd; however, the 5 mg qd group was smaller, and conditions precluding these patients from the recommended regimen may have contributed to worse outcomes. In addition, patients who were able to continue ruxolitinib treatment for 8–14 days were more likely to achieve clinical improvement compared with 7 days of treatment. Overall, fewer than half of patients experienced SAEs, and only 2.6% had suspected ruxolitinib‐related SAEs, without apparent dose dependence. Most SAEs were generally consistent with severe COVID‐19 infection.

Mixed survival benefit has been reported with tocilizumab (IL‐6 inhibitor) in patients with severe COVID‐19 [8, 9], suggesting that selective JAK inhibition may be more effective than blocking individual cytokines for treating COVID‐19–associated cytokine storm [3]. Although the phase 3 RUXCOVID [10] and RUXCOVID‐DEVENT [11] studies did not meet their primary endpoints, clinical activity was observed in some patients. Taken together with phase 3 studies of the JAK1/JAK2 inhibitor baricitinib [12, 13, 14] and ruxolitinib studies [10, 11, 15], this EAP suggests that ruxolitinib may offer clinical status improvements for some patients with the most severe manifestations of COVID‐19.

A strength of this EAP was that patients requiring and not yet requiring mechanical ventilation were included. Interpretation of results was limited by the study design (i.e., nonrandomized, unblinded, and noncontrolled).

In conclusion, some patients with COVID‐19–associated cytokine storm treated with ruxolitinib experienced clinical status improvement. No new safety signals were identified and no thrombotic SAEs were reported. Findings support using bid (vs. qd) dosing when tolerated. Together with ruxolitinib and baricitinib phase 3 studies, these data highlight the need for randomized controlled trials to further assess clinical benefit and identify patients who respond to JAK inhibition in severe COVID‐19.

## Author Contributions

J.W. and N.J. were involved in direct patient management and data collection. S.L.‐J. was involved in the conduct of the study and data analysis. J.W., N.J., J.E.H.‐M., and P.S. contributed to the study design and data analysis. All authors participated in manuscript preparation and manuscript review.

## Ethics Statement

Institutional review boards of participating centers approved the protocol; the trial was conducted per the Declaration of Helsinki and all local regulatory requirements.

## Consent

Patients, their legally authorized representative, and/or legal guardian provided written informed consent prior to study enrollment.

## Conflicts of Interest

JW and NJ have no conflicts of interest to disclose. SL‐J, JEH‐M, and PS are employees and shareholders of Incyte.

## Supporting information


**Table S1.** 9‐Point Ordinal Clinical Scale for Clinical Improvement.
**Table S2.** Patient Demographics and Baseline Clinical Characteristics.
**Table S3.** Treatment‐Related SAEs.
**Table S4.** Medically Significant SAEs Reported in >1 Patient.
**Figure S1.** Physician Assessment of Clinical Benefit.

## Data Availability

Incyte Corporation (Wilmington, DE, USA) is committed to data sharing that advances science and medicine while protecting patient privacy. Qualified external scientific researchers may request anonymized datasets owned by Incyte for the purpose of conducting legitimate scientific research. Researchers may request anonymized datasets from any interventional study (except Phase 1 studies) for which the product and indication have been approved on or after 1 January 2020 in at least one major market (e.g., US, EU, and JPN). Data will be available for request after the primary publication or 2 years after the study has ended. Information on Incyte's clinical trial data sharing policy and instructions for submitting clinical trial data requests are available at https://www.incyte.com/Portals/0/Assets/Compliance%20and%20Transparency/clinical‐trial‐data‐sharing.pdf?ver=2020‐05‐21‐132838‐960.
