# Supplementary material for: Ruxolitinib for Emergency Treatment of COVID‐19–Associated Cytokine Storm: Findings From an Expanded Access Study
Source: Clin Respir J. 2025 Apr 8;19(4):e70050. doi: 10.1111/crj.70050 (PMC11976455; doi:10.1111/crj.70050)
Supplement: Supplementary file 1 — Table S1. 9‐Point Ordinal Clinical Scale for Clinical Improvement. Table S2. Patient Demographics and Baseline Clinical Characteristics. Table S3. Treatment‐Related SAEs. Table S4. Medically Significant SAEs Reported in >1 Patient. Figure S1. Physician Assessment of Clinical Benefit. [file CRJ-19-e70050-s001.docx]

**Ruxolitinib for Emergency Treatment of COVID-19–Associated Cytokine Storm: Findings From an Expanded Access Study**

Jeffrey Weinstein, MD, FIDSA, CPE,^1^ Nikhil Jagan, MD, FCCP,^2^ Shawnta Lorthridge-Jackson, MBA,^3^ J.E. Hamer-Maansson, MSPH,^3^ Peg Squier, MD, PhD^3^

^1^Kettering Health Network, Dayton, OH, USA; ^2^Creighton University School of Medicine, Omaha, NE, USA; ^3^Incyte Corporation, Wilmington, DE, USA

# **Supplementary Information**

[Supplementary Methods 2](#_Toc184883427)

[Table S1. 9-Point Ordinal Clinical Scale for Clinical Improvement 3](#_Toc184883428)

[Table S2. Patient Demographics and Baseline Clinical Characteristics 4](#_Toc184883429)

[Table S3. Treatment-Related SAEs 5](#_Toc184883430)

[Table S4. Medically Significant SAEs Reported in >1 Patient 6](#_Toc184883431)

[Supplementary Figure. Physician Assessment of Clinical Benefit 7](#_Toc184883432)

#### **Supplementary Methods**

*Manifestations of Cytokine Storm*

Manifestations of cytokine storm included respiratory rate >24 breaths/min, oxygen saturation <90% on ambient air, need for mechanical ventilation, ARDS, or multiorgan failure.

*Criteria for Serious Adverse Events*

SAEs were: (1) fatal or life-threatening; (2) required inpatient hospitalization or prolongation of existing hospitalization; (3) resulted in persistent or significant disability, incapacity, or substantial disruption of the ability to conduct normal life functions; or (4) constituted a congenital anomaly or birth defect.

#### **Table S1. 9-Point Ordinal Clinical Scale for Clinical Improvement**

| **Patient State** | **Descriptor** | **Score** |
| --- | --- | --- |
| Uninfected | No clinical or virologic evidence of infection | 0 |
| Ambulatory | No limitations of activities | 1 |
|  | Limitation of activities | 2 |
| Hospitalized, mild disease | Hospitalized, no oxygen therapy | 3 |
|  | Oxygen by mask or nasal prongs | 4 |
| Hospitalized, severe disease | Noninvasive ventilation or high-flow oxygen | 5 |
|  | Intubation and mechanical ventilation | 6 |
|  | Ventilation + additional organ support – vasopressors, renal replacement therapy, ECMO | 7 |
| Death | Death | 8 |

ECMO, extracorporeal membrane oxygenation.

#### **Table S2. Patient Demographics and Baseline Clinical Characteristics**

| **Characteristic** | **Ruxolitinib 5 mg bid (n=280)** | **Ruxolitinib 5 mg qd (n=31)** | **All (N=312)^†^** |
| --- | --- | --- | --- |
| Age, median (range), y | 67.0 (21–97) | 74.0 (37–93) | 67.0 (21–97) |
| Sex, n (%) |  |  |  |
| Men | 162 (57.9) | 17 (54.8) | 179 (57.4) |
| Women | 118 (42.1) | 14 (45.2) | 133 (42.6) |
| Baseline status,^‡^ n (%) |  |  |  |
| 3 | 0 | 1 (3.2) | 1 (0.3) |
| 4 | 46 (16.4) | 7 (22.6) | 53 (17.0) |
| 5 | 178 (63.6) | 11 (35.5) | 190 (60.9) |
| 6 | 37 (13.2) | 5 (16.1) | 42 (13.5) |
| 7 | 19 (6.8) | 7 (22.6) | 26 (8.3) |
| Time to treatment after diagnosis, median (range), d | 6.0 (1–31) | 8.0 (2–44) | 6.0 (1–44) |

bid, twice daily; qd, once daily.

^†^ Total includes 1 patient (79-year-old woman with baseline clinical status of 5 and 2 days to treatment following diagnosis) who did not receive ruxolitinib 5 mg qd or bid.

^‡^ 3=Hospitalized, mild disease, no oxygen therapy; 4=hospitalized, mild disease, oxygen by mask or nasal prongs; 5=hospitalized, severe disease, noninvasive ventilation or high-flow oxygen; 6=hospitalized, severe disease, intubation and mechanical ventilation; 7=hospitalized, severe disease, ventilation + additional organ support – vasopressors, renal replacement therapy, extracorporeal membrane oxygenation.

#### **Table S3. Treatment-Related SAEs**

| **Characteristic** | **Ruxolitinib 5 mg bid (n=280)** | **Ruxolitinib 5 mg qd (n=31)** | **All (N=312)^†^** |
| --- | --- | --- | --- |
| Any treatment-related SAE, n (%) | 5 (1.8) | 3 (9.7) | 8 (2.6) |
| Septic shock | 2 (0.7) | 3 (9.7) | 5 (1.6) |
| Anemia | 1 (0.4) | 1 (3.2) | 2 (0.6) |
| Aspergillus infection | 1 (0.4) | 0 | 1 (0.3) |
| Enterobacter infection | 1 (0.4) | 0 | 1 (0.3) |
| Escherichia infection | 0 | 1 (3.2) | 1 (0.3) |
| Hypersensitivity | 1 (0.4) | 0 | 1 (0.3) |
| Infection | 1 (0.4) | 0 | 1 (0.3) |
| Sepsis | 1 (0.4) | 0 | 1 (0.3) |

bid, twice daily; qd, once daily; SAE, serious adverse event.

^†^ Total includes 1 patient who did not receive ruxolitinib 5 mg qd or bid; the patient did not experience any SAEs.

#### **Table S4. Medically Significant SAEs Reported in >1 Patient**

| **Characteristic** | **Ruxolitinib 5 mg bid (n=280)** | **Ruxolitinib 5 mg qd (n=31)** | **All (N=312)^†^** |
| --- | --- | --- | --- |
| Any medically significant SAE, n (%) | 25 (8.9) | 3 (9.7) | 28 (9.0) |
| Acute kidney injury | 4 (1.4) | 1 (3.2) | 5 (1.6) |
| COVID-19 pneumonia | 4 (1.4) | 0 | 4 (1.3) |
| Acute respiratory failure | 3 (1.1) | 0 | 3 (1.0) |
| Arrhythmia | 1 (0.4) | 2 (6.5) | 3 (1.0) |
| Pneumothorax | 3 (1.1) | 0 | 3 (1.0) |
| Respiratory failure | 3 (1.1) | 0 | 3 (1.0) |
| ARDS | 2 (0.7) | 0 | 2 (0.6) |
| Anemia | 1 (0.4) | 1 (3.2) | 2 (0.6) |
| Pneumonia | 2 (0.7) | 0 | 2 (0.6) |

ARDS, acute respiratory distress syndrome; bid, twice daily; qd, once daily; SAE, serious adverse event

^†^ Total includes 1 patient who did not receive ruxolitinib 5 mg qd or bid; the patient did not experience any SAEs.

#### **Supplementary Figure. Physician Assessment of Clinical Benefit**

**
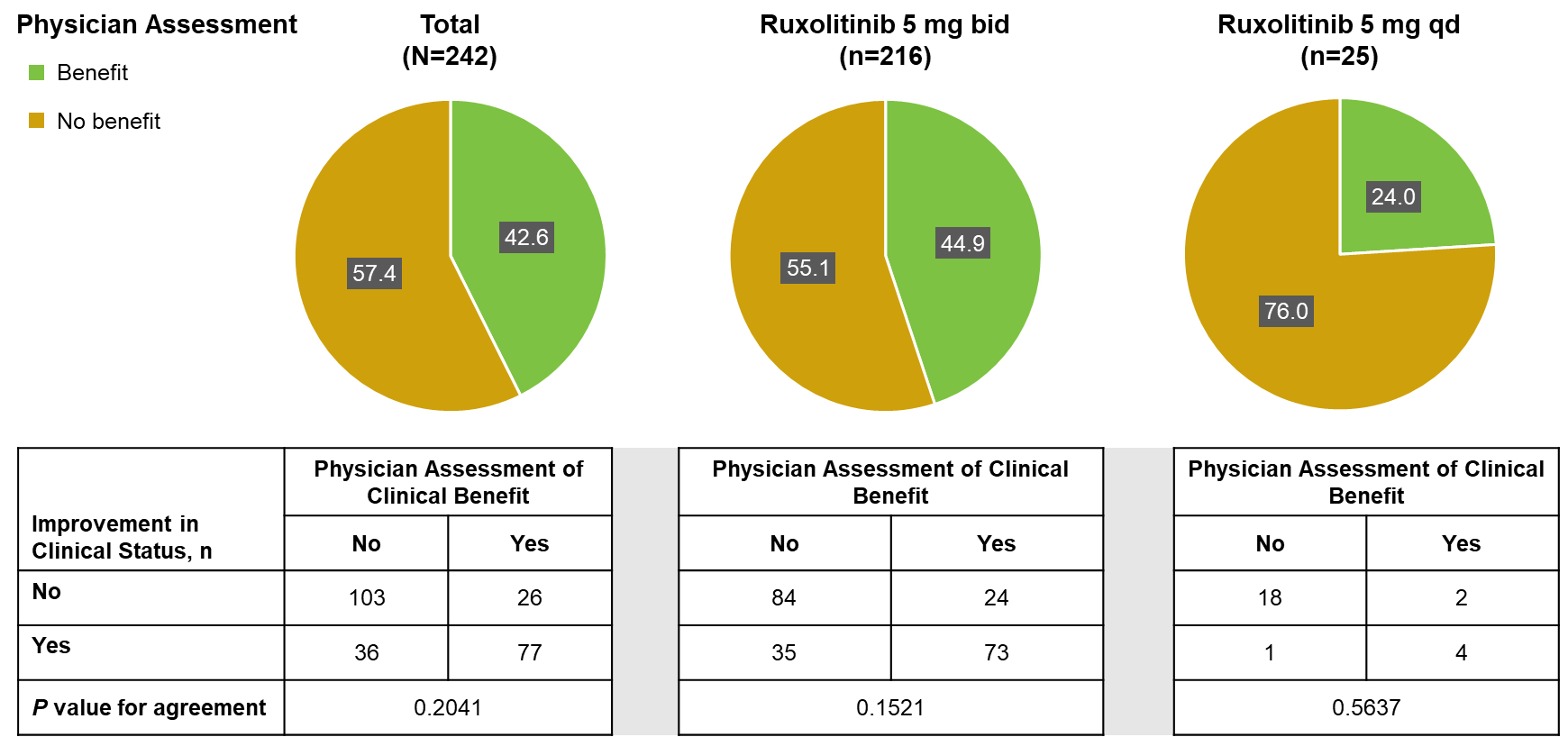
**bid, twice daily; qd, once daily. A total of 242 patients had physician evaluations of clinical benefit. Agreement between physician assessment of clinical benefit and improvement in clinical status (≥1 point per the 9-point ordinal scale) is shown.
